# Attractiveness of volatiles from different body parts to the malaria mosquito *Anopheles coluzzii* is affected by deodorant compounds

**DOI:** 10.1038/srep27141

**Published:** 2016-06-01

**Authors:** Niels O. Verhulst, Berhane T. Weldegergis, David Menger, Willem Takken

**Affiliations:** 1 Laboratory of Entomology, Wageningen University, P.O. Box 8031, 6700 EH Wageningen, the Netherlands

## Abstract

Mosquitoes display biting preferences among different sites of the human body. In addition to height or convection currents, body odour may play a role in the selection of these biting sites. Previous studies have shown that skin emanations are important host-finding cues for mosquitoes. In this study, skin emanations were collected from armpits, hands and feet; the volatile profiles were analysed and tested for their attractiveness to the malaria mosquito *Anopheles coluzzii*. Skin emanations collected from armpits were less attractive to *An. coluzzii* compared to hands or/and feet. The difference may have been caused by deodorant residues, which were found in the armpit samples and not in those of hands and feet. In a subsequent experiment, volunteers were asked to avoid using skincare products for five days, and thereafter, no differences in attractiveness of the body parts to mosquitoes were found. The detected deodorant compound isopropyl tetradecanoate inhibited mosquito landings in a repellent bioassay. It is concluded that the volatiles emanated from different body parts induced comparable levels of attraction in mosquitoes, and that skincare products may reduce a person’s attractiveness to mosquitoes.

Human body odours play an important role in the host-seeking behaviour of mosquitoes^1^. Mosquito species like the malaria mosquito *An. coluzzii* (formerly *An. gambiae sensu stricto* molecular form M) and the yellow fever mosquito *Aedes aegypti* are effective disease vectors as they mainly bite humans^2^. These anthropophilic mosquitoes use human specific odours, next to more general cues like body heat and carbon dioxide, to find their hosts^1^. The influence of human body odour on the attractiveness of individuals to mosquitoes has been studied in detail^3^,^4^. Humans vary in their attractiveness to mosquitoes, and the differences are relatively stable over time^3^,^5^. The analysis of the body odours of individuals with varying attractiveness has led to the identification of several attractive and repellent volatiles that can be used as novel vector control tools^3^,^6^.

Differences in attractiveness to mosquitoes also occur between body sites and vary considerably between mosquito species. *Aedes aegypi*, *Ae. simpsoni* and *An. atroparvus,* for example, prefer to bite around the head and shoulder^7^,^8^,^9^, while *Culex quinquefasciatus* does not seem to prefer any specific body part^8^. *Anopheles gambiae s.s., An. arabiensis* and *An. funestus* are important malaria vectors and bite most frequently on the feet and ankles^7^,^10^,^11^, however, when people lay down, this preference disappears and they may bite anywhere on the body except the head^10^,^11^. Dekker *et al.*^11^ have suggested that mainly convection currents and partly host odours guide mosquitoes to the feet and ankles, while Braack *et al.*^10^ attribute the selection of biting sites mainly to the height above ground^10^,^12^.

The underlying mechanisms behind the variation in attractiveness between individuals to mosquitoes have been studied in detail and have shown that body odours play an important role as well^5^,^13^,^14^,^15^. Less is known, however, about the mechanisms behind the variation in attractiveness of different body regions of the same individual. Washing the feet with antibacterial soap diverts *An. gambiae s.s.* to other body parts^7^ and worn socks are highly attractive to this mosquito species under both laboratory and field conditions^16^,^17^,^18^. This suggests that human volatiles play a role in the selection of biting sites.

Several studies have investigated the volatile profiles from the human body, resulting in more than 500 compounds that have already been reported^19^,^20^,^21^,^22^. However, it is difficult to compare the volatile profiles of the different body parts due to the use of different sampling and processing techniques across these studies^23^. The volatile composition of the skin depends on the type and number of sweat glands and the bacteria that thrive on the products of these glands^24^,^25^. Bacteria convert long chain non-volatile compounds into short chain volatile compounds that are attractive to mosquitoes^26^. In addition, the attractiveness of human skin emanations to mosquitoes is correlated with the bacterial diversity and composition of the skin^15^.

In the current study it was tested if the volatiles collected from feet, hand and armpit varied in their attractiveness to the malaria mosquito *An. coluzzii*. In addition, the volatile profiles of these body parts were analysed to identify the compounds that could mediate differences in attractiveness to *An. coluzzii*. In a first experiment the volatiles of the three body parts of eight individuals were tested for their attractiveness to *An. coluzzii* in a dual choice olfactometer against a standard of ammonia^5^,^15^. In a second experiment the volatiles of the three body parts were tested directly against each other pairwise. Analysis of the volatile profiles from the first series of experiments revealed some exogenous compounds that could have explained the reduced attractiveness of certain body parts. Therefore, the protocols for the individuals participating in the second series of experiments were rather strict and the exogenous compounds were tested for potential repellent effects on *An. coluzzii*. In addition, the ability of the exogenous compounds to affect attractiveness indirectly, by inhibiting skin bacteria and thereby volatile production was investigated.

## Results

### Volatile analysis

**Figure 1:**
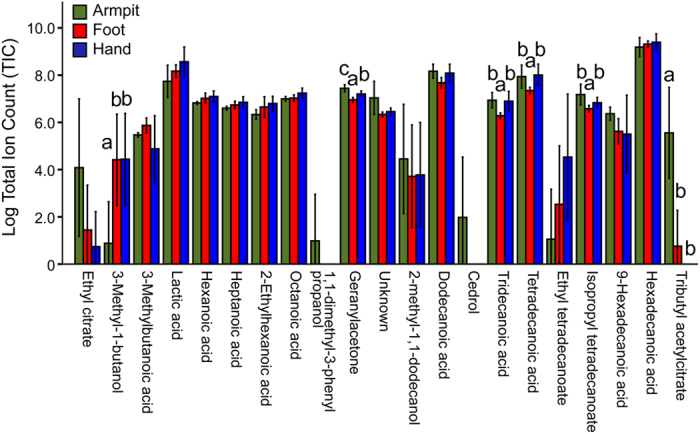
Abundances (log) of compounds identified in skin emanations from different body parts. Individuals did not shower of use skincare products for a period of 24 hr before sampling. Different letters indicate significant differences in the abundance of a compound between body parts (P < 0.05). Error bars indicate standard errors of the mean.

On the cotton pads with skin emanations of individuals that were not allowed to use soaps or perfume/s for 24 hr, the abundance of 21 identified compounds as determined by their peak areas were significantly higher than from the control pads. The abundance of six compounds was significantly different between the different body parts (ANOVA, P < 0.05, Fig. 1). The abundance of 3-methyl-1-butanol was significantly lower on pads from armpits than on pads from feet or hands and the abundance of tributyl acetylcitrate was significantly higher on the armpit pads than on pads from feet or hands. The abundance of geranylacetone, tridecanoic acid, tetradecanoic acid and isopropyl tetradecanoate was significantly lower in the foot samples (Fig. 1).

**Figure 2:**
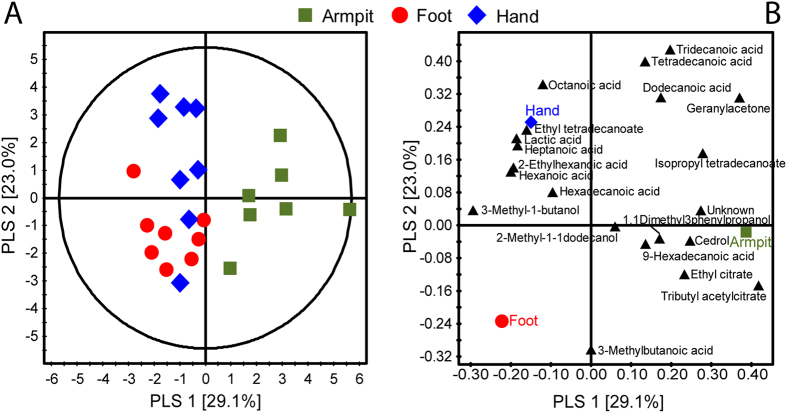
Projection to latent structures-discriminant analysis (PLS-DA) score plot (**A**) and loading plot (**B**) of volatile patterns of armpit, foot and hand. Individuals did not use skincare products for 24 hr before sampling. Volatiles closer to the armpit, hand or foot in the plot are more correlated to their corresponding body parts. Percentage variation explained for each PLS-DA axis is given in parentheses.

A Partial Least Square Discriminant Analysis (PLS-DA) could clearly separate the armpit samples from the samples of the other body parts based on the quantitative values of the volatiles with small degree of overlap between the hand and foot samples (R^2^X = 0.604, R^2^Y = 0.736, Q^2^ = 0.490; Fig. 2). Except for 3-methylbutanoic acid, no volatiles were highly correlated with foot odour. Volatiles that were most influential for the separation of the different body parts were tributyl acetylcitrate, geranylacetone, 3-methyl-1-butanol, 3-methylbutanoic acid and tridecanoic acid with VIP (Variable Importance in the Projection) values >1.

Isopropyl tetradecanoate, tributyl acetylcitrate and tridecanoic acid are commonly used in deodorants and may have influenced the mosquito bioassay, non-host associated compounds^27^. Therefore, the protocols for the individuals participating in the second series of experiments were made more strict and individuals were not allowed to use skin products for five days. Now, the abundance of the deodorant compounds isopropyl tetradecanoate and tributyl acetylcitrate was not significantly different from the control pads and the compound was only found in three samples (ANOVA, P < 0.05). Tridecanoic acid is also naturally released from the human skin^28^ and was still detected in the samples (Supplementary Fig. S1). Volatiles of which the abundance was different between the control and worn pads were generally present in higher abundance in the hand samples (Supplementary Fig. S1), which was confirmed by the PLS-DA analysis (R^2^X = 0.566, R^2^Y = 0.596 Q^2^ = 0.463; Supplementary Fig. S2).

### Bioassay mosquito attraction

**Figure 3:**
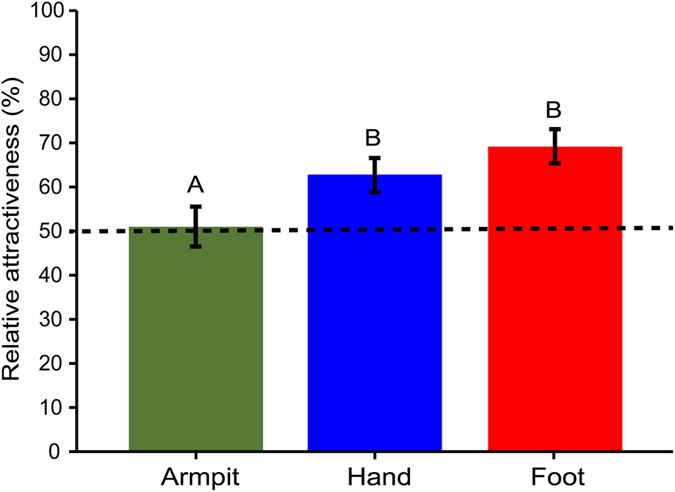
Relative attractiveness (back-transformed, GLM) of the volatiles from different body parts of individuals to *Anopheles coluzzii*. Individuals were not allowed to shower and use skincare products for 24 hours before sampling. Error bars indicate standard errors of the mean. Different letters indicate significant differences (GLM, y = x_1_ + x_2_*individual + x_3_*body part, P < 0.05).

**Figure 4:**
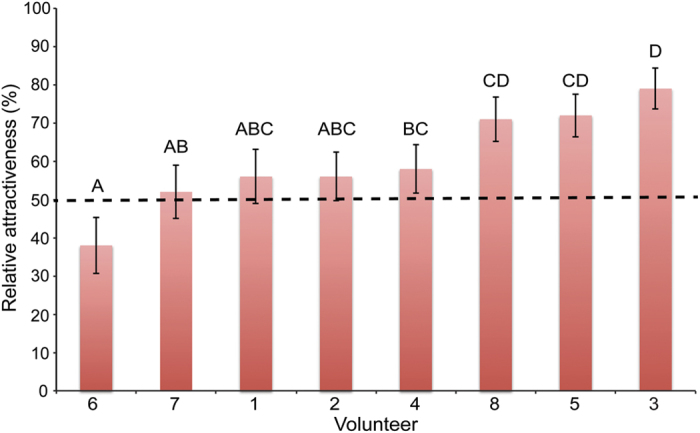
Relative attractiveness (back-transformed, GLM) of eight individuals to *Anopheles coluzzii*. Individuals were not allowed to shower and use skincare products for 24 hours before sampling. Error bars indicate standard errors of the mean. Different letters indicate significant differences (GLM, y = x_1_ + x_2_*individual + x_3_*body part, P < 0.05).

The relative attractiveness (RA) of mosquitoes to collected volatiles from individuals that were not allowed to use soaps or perfumes for 24 hr was significantly influenced by both individuality and their body part of which the volatiles were collected (GLM, P = 0.001 and P = 0.009, respectively, RA = x_1_ + individual*x_2_ + body part*x_3_, Supplementary Table 1). Other factors, like position of treatment in the olfactometer, layer of the olfactometer, temperature and humidity did not have a significant effect on the RA and no interactions were found (GLM, P > 0.05). The RA (back-transformed, GLM) to volatiles collected from the armpit (51%) was significantly lower than the RA to volatiles collected from the hand (63%, GLM, P = 0.038) and from feet (69%, GLM, P = 0.002, Fig. 3). The volatiles of all individuals attracted more mosquitoes than the control of ammonia, except for the volatiles from individual #6 (Fig. 4). The RA of the volatiles to mosquitoes differed significantly between individuals, ranging from a RA of 38% for individual #6 to 79% for individual #3 (Back-transformed, GLM, P < 0.05, Fig. 4, Supplementary Table 1).

**Figure 5:**
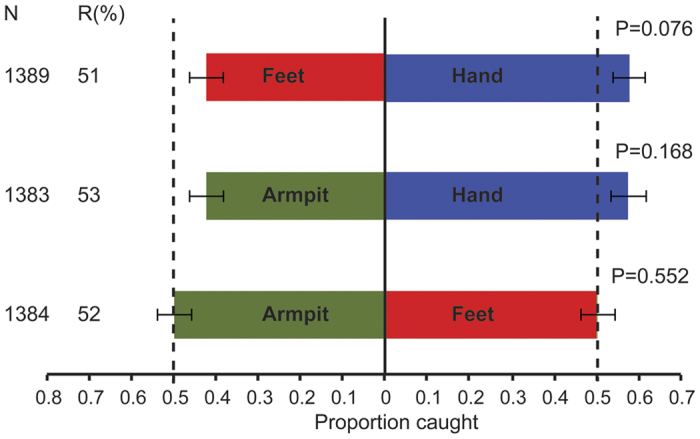
Dual-choice essay for the response of *Anopheles coluzzii* to different body parts. Individuals did not use skincare products for five d prior to sampling. N = number of mosquitoes released. R = Trap entry response expressed as the number of mosquitoes trapped in both trapping devices divided by the number of mosquitoes released. Error bars indicate standard errors of the mean. P-values indicated (GLM).

The second group of individuals was asked not to use skin products for five d to exclude the effect of these skincare products on the volatile profiles. No significant differences were found between the volatiles from different body parts of this group of individuals when tested directly against each other in the olfactometer (GLM, P > 0.05, Fig. 5, Supplementary Table S2). The individual from which the volatiles were collected influenced the direct comparison between foot and armpit volatiles and was included in the model for this comparison (GLM, P = 0.004). Other factors did not have a significant effect on the proportion of mosquitoes caught and no interactions were found (GLM, P > 0.05).

### Repellency experiment

The purpose of the repellency experiment was to identify whether any of the three candidate compounds, tridecanoic acid, isopropyl tetradecanoate or tributyl acetylcitrate, had a repellent effect on *An. coluzzii*. DEET (N,N-diethyl-meta-toluamide) is one of the best-known mosquito repellents and was used as a positive control. Each candidate compound was tested individually in three concentrations.

**Figure 6:**
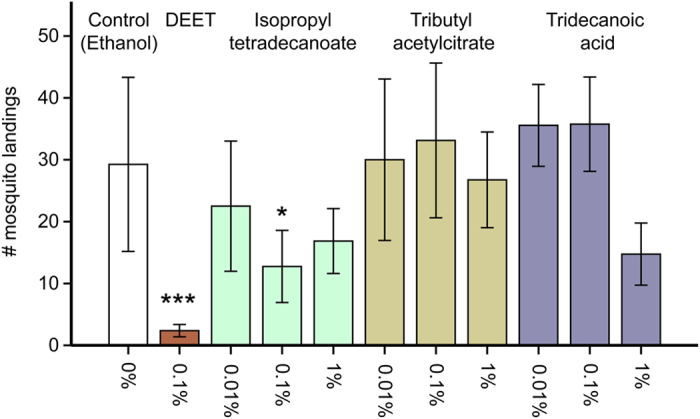
Effects of the candidate repellents on *Anopheles coluzzii* landings. Bars show the mean number of landings made by a group of 10 females during 8 min. Error bars indicate the standard error of the mean: ***P < 0.001, *P < 0.05, ANOVA followed by Dunnet’s post-hoc test compared to the control.

On average there were 29.25 landings of 10 mosquitoes in 8 min when the control (ethanol) was tested and only 2.38 landings when 0.01% DEET solution was tested (91.9% reduction, ANOVA, P < 0.001, Fig. 6), which was in accordance with initial pilot experiments (Supplementary Fig. S3). All concentrations of isopropyl tetradecanoate reduced the number of landings; the difference between the control and 0.1% isopropyl tetradecanoate was significant and reduced the number of landings by 56.4% to 12.75 (ANOVA, P = 0.040, Fig. 6). Tributyl acetylcitrate did not reduce the number of landings of *An. coluzzii* on the synthetic odour blend (ANOVA, P > 0.05). Although, tridecanoic acid reduced the number of landings when the highest concentration of 1% was tested, this reduction was not significant (P = 0.080, Fig. 6).

### Skin bacterial inhibition experiment

It was tested whether any of the three candidate compounds, tridecanoic acid, isopropyl tetradecanoate or tributyl acetylcitrate had an inhibitory effect on the most common skin bacteria, *Staphylococcus epidermidis*, that produces volatiles attractive to *An. coluzzii*^29^,^30^. No inhibitory effect by any of the compounds of interest was found (ANOVA, P > 0.05, Supplementary Fig. S4). When only water was added to the TSA plates, on average 310 colony forming units (cfu’s) of *S. epidermidis* were counted, which was not significantly different from the number of cfu’s on the plates where ethanol or any of the tested compounds were added (ANOVA, P > 0.05, Supplementary Fig. S5).

## Discussion

In the initial experiment with volatiles collected from feet, hand and armpit of eight individuals, armpit odour was significantly less attractive to *An. coluzzii* compared to hand or feet odour. However, volatile analysis of these samples indicated that some residues of skincare products were still present, especially in the armpit samples, even though individuals did not use fragranced products from 24 h. before sampling. Repellency tests with three of these compounds that were suspected to decrease the attractiveness of armpit samples showed that isopropyl tetradecanoate reduced the number of landings significantly by 56.4% compared to the control. Isopropyl tetradecanoate is used in cosmetics as an emulsifier or solubilizer and can be found in creams and deodorants^27^. The effect of these skincare products on the attractiveness of mosquitoes seemed to be confirmed by the second experiment for which volunteers were asked to refrain from using perfumed products for five days. In this experiment the abundance of the compounds isopropyl tetradecanoate and tributyl acetylcitrate on the worn cotton pads was not significantly different from the control pads (P > 0.05), which suggests that these compounds were indeed deodorant residues as hypothesized.

The three body sites selected for this study vary considerably in temperature, humidity and number and type of skin glands^25^. Apocrine sweat glands are very dominant in armpits, but not found on hands or feet^31^. Apocrine glands are a major source of sweat production and assumed to play a role in human pheromone production^32^. Sebaceous skin glands produce sebum and are more abundant in armpits than on hands and feet while eccrine glands are abundant on both hands and feet and release sweat for cooling of the body^25^. Each of these body sites has its own microbial composition, with Staphylococcus being dominant on the feet, Corynebacterium together with Staphylococcus in the armpit and Propionibacterineae on the hands^33^,^34^,^35^. These differences result in a very diverse odour profiles emitted from each body part as shown in this and previous studies^23^,^36^. Nevertheless, no differences were found between the attractiveness of mosquitoes to the volatile samples from different body parts. This result suggests that mainly convection currents or height above ground determine biting site selection and that host odours that cause attraction are probably produced across the entire body. Previous studies have indicated that these volatiles attractive to mosquitoes are relatively stable over time^5^ and that the origin of these volatiles is, at least partly, genetically determined^37^,^38^.

The use of cotton pads for both behavioural experiments and volatile analysis was developed for this study and proved to be a reliable method of testing the attractiveness of skin emanations. Previous studies analysed human odour profiles by solvent extraction, dynamic headspace adsorption or solid phase micro-extraction (SPME)^23^. In this study, the use of thermal desorption (TD) directly on the pieces of cotton pads that were also used in the behavioural experiments ensured that the differences in volatile profiles detected by gas chromatography combined with mass spectrometry (GC-MS) and encountered by mosquitoes were minimal. Although, “clean” unused cotton pads release a range of volatiles (this study), clear differences were found in the profiles of clean and worn pads and the PLS-DA analysis could significantly differentiate between the volatile profiles of pads worn on different body parts (Fig. 2 and Supplementary Fig. S2).

Skincare products can be used to mask human malodour e.g. perfumes or reduce malodour by inhibiting bacterial growth. Skin bacteria play an important role in the production of human malodour^39^,^40^, but also in the production of volatiles that attract mosquitoes^41^. The number of *Staphylococcus* spp., for example, is correlated with a person’s attractiveness to mosquitoes^15^ and reducing the number of bacteria on the legs by antibacterial soap diverts mosquitoes to other body parts^7^. Although deodorants and antiperspirants affect the axillary bacterial community^35^,^42^, the three compounds of interest did not reduce the number of *S. epidermidis* on agar plates (Supplementary Fig. S5). From these *in vitro* experiments it appears that the compounds tested do not affect the attractiveness of the individuals for mosquitoes indirectly by causing a decrease in the number of *Staphylococcus* on their skin and thereby the production of attractive volatiles. *In vivo* experiments, in which the skin microbiota and volatile composition monitored before and after skin product use should confirm this.

Little is known about the effects of grooming on the attractiveness of individuals to mosquitoes, although this will certainly play a role. In earlier work a high but consistent variability between individuals in attractiveness to mosquitoes was demonstrated^3^,^5^,^15^. These variations could be explained, among others, by age^43^, skin temperature^44^ or infection with malaria parasites^45^. This study showed that skincare products may have a direct effect on the number of mosquitoes landing. The effects of grooming on the attractiveness of humans to mosquitoes can be studied in more detail by applying known formulas on the skin and monitor mosquito attraction, volatile profiles and skin microbial communities.

The results presented in the current study did not reveal a difference in the attractiveness of *An. coluzzii* to the volatiles collected from feet, hand or armpit. However, the results do indicate that skincare products may reduce a person’s attractiveness to mosquitoes directly by reducing the number of landings instead of indirectly by changing the skin microbiota. Studying the effect of skincare products on an individual’s attractiveness to mosquitoes will lead to a better understanding of the interactions on the skin that play a role in this attraction and may lead to specific products that help to reduce a person’s attractiveness to mosquitoes and thereby the number of bites received.

## Methods

### Mosquitoes

The *An. coluzzii* (renamed from *An. gambiae sensu stricto* molecular M form;^46^) colony at Wageningen University, the Netherlands, originated from Suakoko, Liberia 1987. Mosquitoes were reared according to the methods described previously^47^. Adult mosquitoes were fed every day on human blood through a membrane system with the addition of human odour from a worn sock and 5% CO_2_ to mimic a human host.

### Individuals

The individuals were non-smoking Caucasian males, between 20 and 50 years old. They were requested to refrain from drinking alcohol^48^, eating heavily scented or spicy food like onions, garlic and peppers and to avoid the use of skincare products^5^. In the first experiment, eight individuals, who were asked to follow the rules for 24 h., participated. In addition to this, they were instructed not to shower within these 24 h. and have their last shower without soap^5^. In the second experiment eight (other) individuals participated that were asked to follow these rules for five consecutive days, however, they were allowed to shower until 24 h. before the experiment with perfume free shampoo and shower gel (Neutral, Unilever, The Netherlands). Informed consent was acquired from all subjects prior to participation. The study was in accordance with the experimental protocol that was reviewed by the Medical Ethical Reviewing Committee of Wageningen University (METC-WU). The METC-WU concluded that the study did not fall within the remit of the ‘Medical Research Involving Human Subjects Act’, which means that the researchers are lawfully not obliged to obtain ethical approval from a recognized medical research ethics committee for this particular research.

### Volatile sampling

Skin volatiles were sampled from each individual from the sole of the left foot, left armpit and palm of the left hand. Volatiles were collected on 4 × 12 cm cotton pads (HEMA, The Netherlands), that were cut in two pieces and attached to the different body parts with 8 × 15 cm island plasters (Kruidvat, The Netherlands). Between the two pieces of the cotton pads and the plasters a piece of aluminium foil was placed to prevent contamination of the cotton pads with volatiles from the plaster. Volatiles were collected overnight for eight hours and the cotton pads subsequently stored in 10 mL glass vials at −20 °C until use.

Glass jars were cleaned before use with tap water, demineralized water and 70% ethanol solution and dried in an oven at 150 °C for 24 hours. Cotton pads were cleaned before use with hexane (Merck, Germany) and methanol (Sigma-Aldrich, Germany) and dried in the cleaned glass jars in an oven at 100 °C for two hours.

### Volatile analysis

Of each cotton pad coated with human skin emanations, small samples were taken with sterile forceps at three different places on the pads with a total weight of 32.7 mg. The cotton samples of 32.7 mg were transferred to a glass tube that was placed in an autosampler thermal desorption unit (Ultra 50:50 TD, Markes International Ltd, UK).

Desorption from the cotton samples, separation, detection and identification of volatiles were carried out as described previously by Mweresa *et al.*^49^ with minor modification, where thermal desorption was done at 150 °C for 20 min. and separation of volatiles was using the following GC conditions: oven initial temperature was 40 °C and was immediately raised at 5 °C/min to a final temperature of 280 °C and was kept for 4 min.

Volatile profiles collected on cotton pads were compared to control profiles of clean cotton pads in Xcalibur (Version 2.07, Thermo Scientific, USA). Relative quantification of the compounds was done based on characteristic mass ions for each compound and the expected retention time, characteristic mass and integration settings were inserted in a processing setup and batch-processed^38^. Compounds were identified by comparing mass spectra and retention times with those of authentic reference compounds. Volatile profiles from the second group of individuals were screened for the same compounds that were detected to be more abundant than the control in the first group.

### Behavioural assay to determine mosquito attraction

The attractiveness of the skin emanations from different body parts of the individuals was tested in a three layer dual-choice olfactometer according to the methods described by Verhulst *et al.*^50^. In each trial and each layer of the olfactometer, 30 female *An. coluzzii* were released that were 5–8 d old and only had access to tap water in the 24 h. prior to the experiment. Charcoal-filtered air was heated (27.0 ± 1.2 °C), humidified (>80%) and was led through two trapping devices containing the test odour, into the flight chamber. The airflow at the ports of the trapping devices was maintained at a speed of 0.21 ± 0.02 m/s. Mosquitoes were released and allowed to fly upwind for 15 min. towards two trapping devices^50^.

Cotton pads with skin emanations from the first group of individuals were tested against a standard of ammonia, which is moderately attractive to *An. coluzzii*^5^,^51^. Clean air was pumped into the trapping devices containing either cotton pads with skin emanations or gaseous ammonia (136 ppm) with a control cotton pad^51^. In each round of 15 min., the cotton pads of the three different body parts of one individual were tested in the three layers of the olfactometer against ammonia. Each body part of each individual was tested six times on different days and alternated between right and left ports of an olfactometers to rule out any positional and day effects.

Cotton pads with skin emanations from the different body parts of the second group of individuals were tested against each other, for each individual. Because three body parts were sampled (foot, armpit, hand), three different choice experiments were run for each individual. All combinations for each individual were tested on the same day and repeated six times on different days. Treatments were alternated between right and left ports of the three different layers of the olfactometer to rule out any positional or layer effects.

### Repellency experiment

#### Candidate compounds

Tridecanoic acid, isopropyl tetradecanoate and tributyl acetylcitrate were dissolved in ethanol at 0.01, 0.1 and 1% levels and applied on a 15 × 15 cm piece of cotton net fabric (see below). The negative control was consisted of a fabric treated with ethanol only, while the positive control was treated with 0.1% DEET (N,N-diethyl-meta-toluamide). A pilot experiment had served to identify at which concentration of DEET the number of landings would be significantly reduced, while still being high enough to allow the identification of stronger repellents among the compounds of interest (Supplementary Fig. S3).

All compounds tested were applied on net fabric (Leno structure, 65 g/m^2^, provided by Utexbel, Belgium) by a simple dip-and-dry method. The day before the experiment took place, the net fabric was impregnated by placing it into 15 mL solution in a 25 mL blue-cap reaction tube. It was stored overnight at room temperature and the next morning the net material was dried on a steel rack in a flow cabinet for 15 min. Each 15 × 15 cm piece of fabric was used for two successive replicates of the same treatment, after which it was discarded. A total of eleven different treatments were tested, eight times each and in random order (in sets of two successive replicates).

#### Bioassay mosquito attraction

The bioassay described by Menger *et al.*^52^ was used to quantify the repellent efficacy of the selected compounds. It was set up in a climate-controlled room (24 ± 1 °C, RH 60–75%). Central to the bioassay was a landing stage to which mosquitoes were attracted. It consisted of a heated circular plateau (diameter 15 cm) that presented a five-compound attractive odour blend and was positioned underneath the gauze bottom of a flight chamber^52^,^53^,^54^. The temperature in the centre of the landing stage was kept at 34 ± 2 °C, comparable to the temperature of human skin, causing the mosquitoes to land.

Repellency was measured by releasing ten female mosquitoes into flight chamber. After two min. of acclimatization time, the number of landings on the fabric covering the landing stage was counted during eight min. A landing was defined as the total period during which a mosquito maintained contact with the landing stage. Walking/hopping around or on the landing stage as well as short (<1 s) take offs immediately followed by landing again were included in one landing. A new landing was recorded when a mosquito had left the stage for more than 1 s before landing again. Landings shorter than 1 s during which no probing took place were ignored. Each treatment was tested eight times with ten mosquitoes.

### Skin bacterial inhibition experiment

The three candidate compounds were tested at concentrations of 0.01 and 1% in ethanol and DEET at 1% in ethanol. Sterile water and 100% ethanol were included as controls. 100 μL of each of the treatment was spread on tryptic soy agar (TSA) (Becton & Dickinson, The Netherlands) plates and dried for 30 min. under sterile conditions. Next, each plate was inoculated with 100 μL *S. epidermidis* (strain 20044, DSMZ, Germany) solution of approximately 3.10*10^3^ cfu/mL water. Plates were incubated for 48 hours at 34 °C and cfu’s counted. Each of the treatments was tested three times.

### Data analysis

All data were analysed using IBM SPSS Statistics 22, unless stated otherwise. All effects were considered significant at P < 0.05 and corrected for multiple comparisons as indicated for the different tests. Data from the volatile analysis, repellency and bacteria inhibition experiment were checked for normality before an Analysis of Variance (ANOVA) was performed.

#### Volatile analysis

Peak areas of identified volatiles in the chromatograms were first log transformed; next, compounds with an abundance that was not significantly higher than the control as determined by Analysis of Variance (ANOVA) followed by a post-hoc test with LSD correction were removed from the dataset.

Differences in the abundances of volatiles of the three body parts were analysed by an analysis of Variance followed by a post-hoc test with LSD correction. In addition, a Partial Least Square Discriminant Analysis (PLS-DA, SIMCA-P 12.0, Umetrics, Sweden), in which class membership of the observations is predefined, was used to separate the three body parts based on the body odour profiles. Creating a loading plot of the PLS-DA allowed visualizing which volatiles correlate to the different body parts^38^,^55^. Before PLS-DA was carried out, the peak areas were log transformed, mean centred and scaled to unit variance. The number of significant PLS-DA components was determined by cross-validation^55^.

#### Behavioral experiments

Data was analysed with a generalized linear model (GLM, Binomial, logit link function, dispersion estimated) to investigate differences in relative attractiveness between the different body parts and between individuals. The relative attractiveness (RA) was expressed as the fraction of mosquitoes caught in one trapping device divided by the total number of mosquitoes trapped in the two trapping devices together^5^.

The RA was used to investigate whether: the volatiles from the different body parts differed in their attractiveness when compared to ammonia (experiment 1)^5^, and to investigate if the volatiles from one body part collects more mosquitoes than the other, when tested directly against each other, i.e. differed from a 50:50 distribution (experiment 2)^5^,^15^.

The effects of individuals, position of treatment, layer of the olfactometer, temperature and humidity were tested and fitted as parameters in the model when significant. Differences between treatments were tested using pairwise comparisons with Least Square Differences (LSD) correction.

#### Repellency experiment

The number of landings on the treated fabrics were compared to the negative control, using ANOVA followed by Dunnet’s post-hoc test to determine significant reductions.

#### Bacterial inhibition experiment

The number of colony forming units on the treated agar plates were compared to the control plate with ethanol only, using ANOVA followed by Dunnet’s post-hoc test to determine significant reductions.

## Additional Information

**How to cite this article**: Verhulst, N. O. *et al.* Attractiveness of volatiles from different body parts to the malaria mosquito *Anopheles coluzzii* is affected by deodorant compounds. *Sci. Rep.* **6**, 27141; doi: 10.1038/srep27141 (2016).

## Supplementary Material

Supplementary Information
