# Supplementary material for: Attractiveness of volatiles from different body parts to the malaria mosquito Anopheles coluzzii is affected by deodorant compounds
Source: Sci Rep. 2016 Jun 1;6:27141. doi: 10.1038/srep27141 (PMC4890431; doi:10.1038/srep27141)
Supplement: Supplementary Information [file srep27141-s1.pdf]

Supplementary Online Material Verhulst et al.

Attractiveness of volatiles from different body parts to the malaria mosquito *Anopheles coluzzii* is affected by deodorant compounds

Niels O. Verhulst[1], Berhane T. Weldegergis[1], David Menger[1], Willem Takken[1]

[1]Laboratory of Entomology, Wageningen University, P.O. Box 8031, 6700 EH Wageningen, the Netherlands

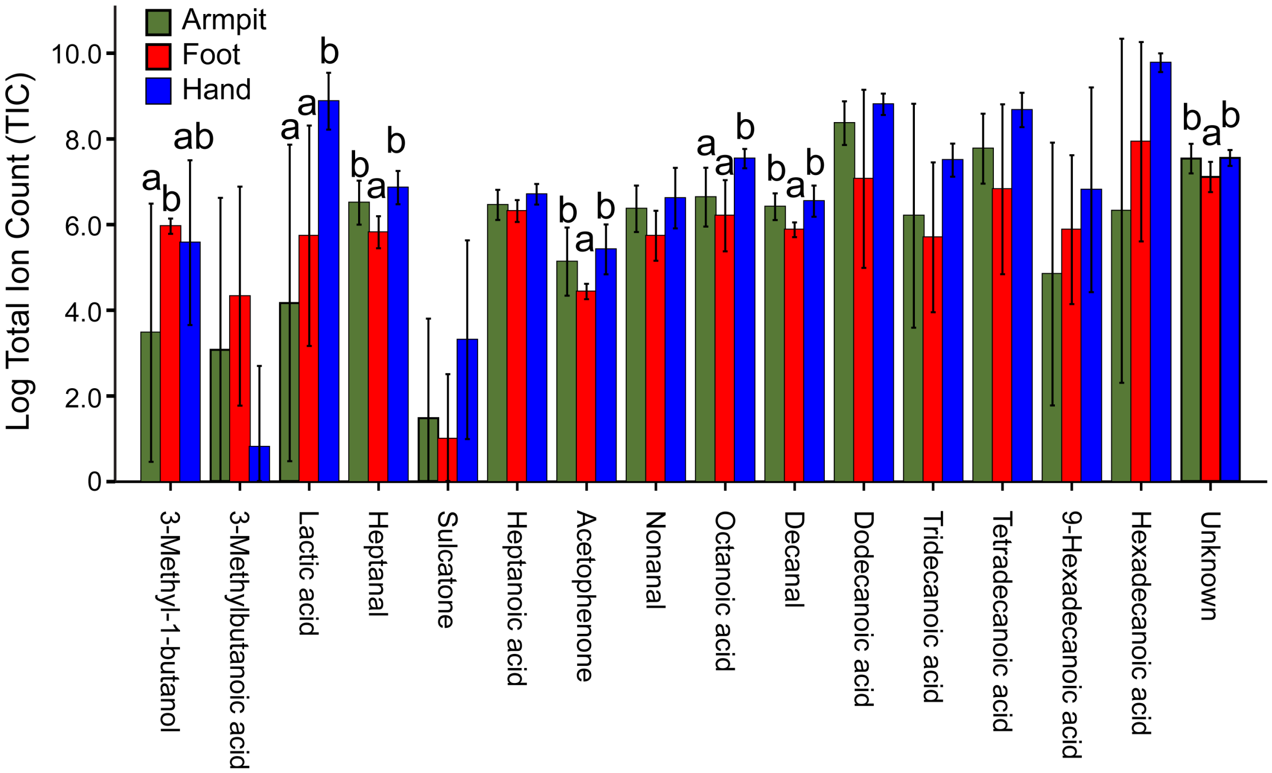

Supplementary Figure S1. Abundances (log) of compounds identified in skin emanations from different body parts.

Individuals were not allowed to use skincare products for five days before sampling. Different letters indicate significant differences in the abundance of a compound between body parts ($P < 0.05$). Error bars indicate standard errors of the mean.

Supplementary Figure S2

Projection to latent structures-discriminant analysis (PLS-DA) score plot (A) and loading plot (B) of volatile patterns of armpit, foot and hand.

Individuals were not allowed to use skincare products for five days before sampling. Volatiles closer to the armpit, hand or foot in the plot are more correlated to either body part. Percentage variation explained for each PLS-DA axis is given in parentheses.

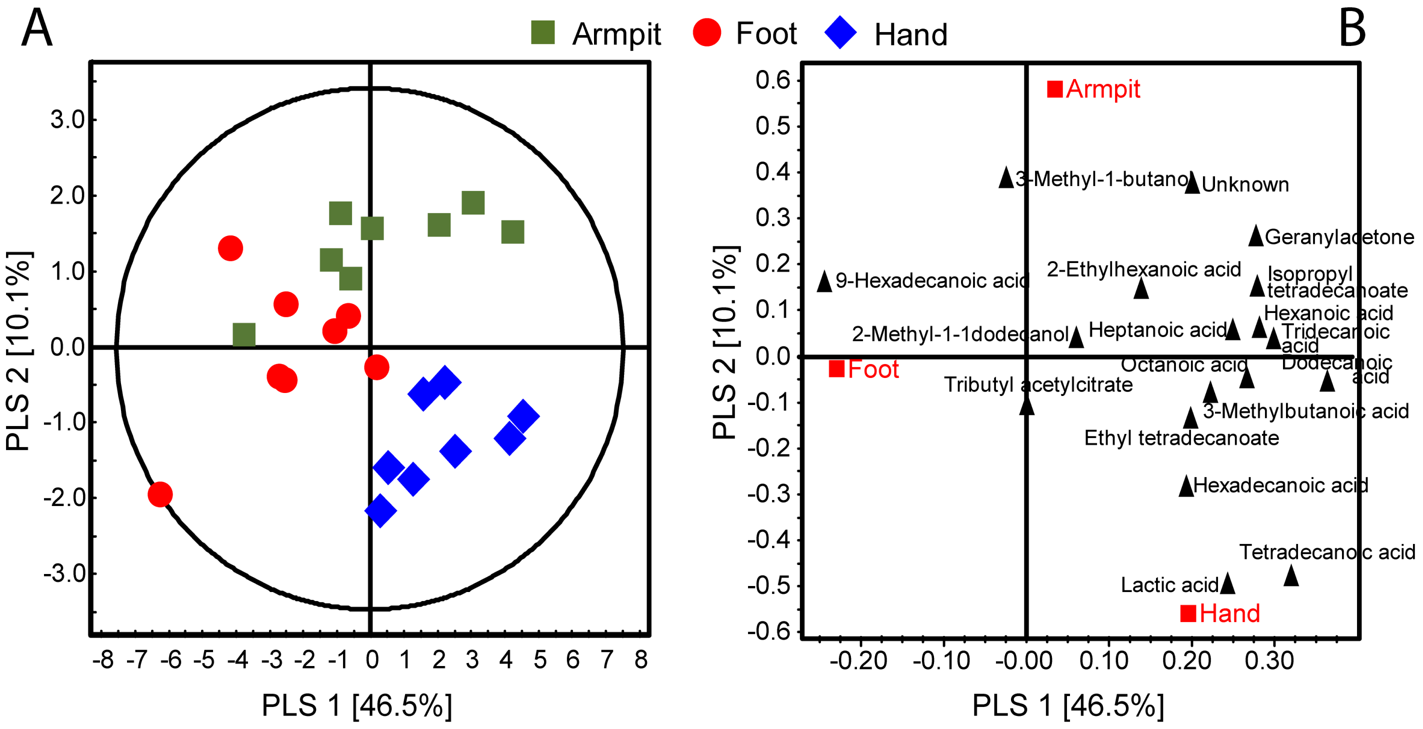

Supplementary Figure S3 Effects of three concentrations of DEET on *Anopheles colluzi* landings. Bars show the mean number of landings made by a group of 10 females during 8 min (N=8). Error bars indicate the standard error of the mean: ***: P <0.001, ANOVA followed by Dunnet's post-hoc test compared to the control.

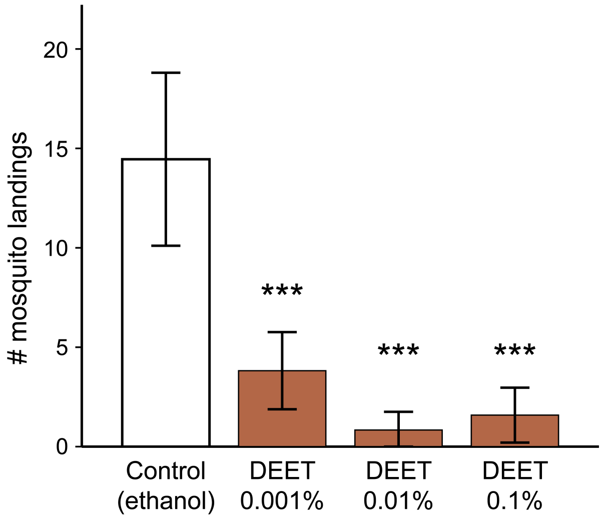

Supplementary Figure S4

Effects of candidate compounds on the growth of the skin bacteria *Staphylococcus epidermidis*.

TSA plates with or without the candidate compounds were inoculated with *S. epidermidis* and number of cfu's counted after 24h. N = 3. Error bars indicate the standard error of the mean. No significant differences were found; ANOVA followed by Dunnet's post-hoc test compared to the control.

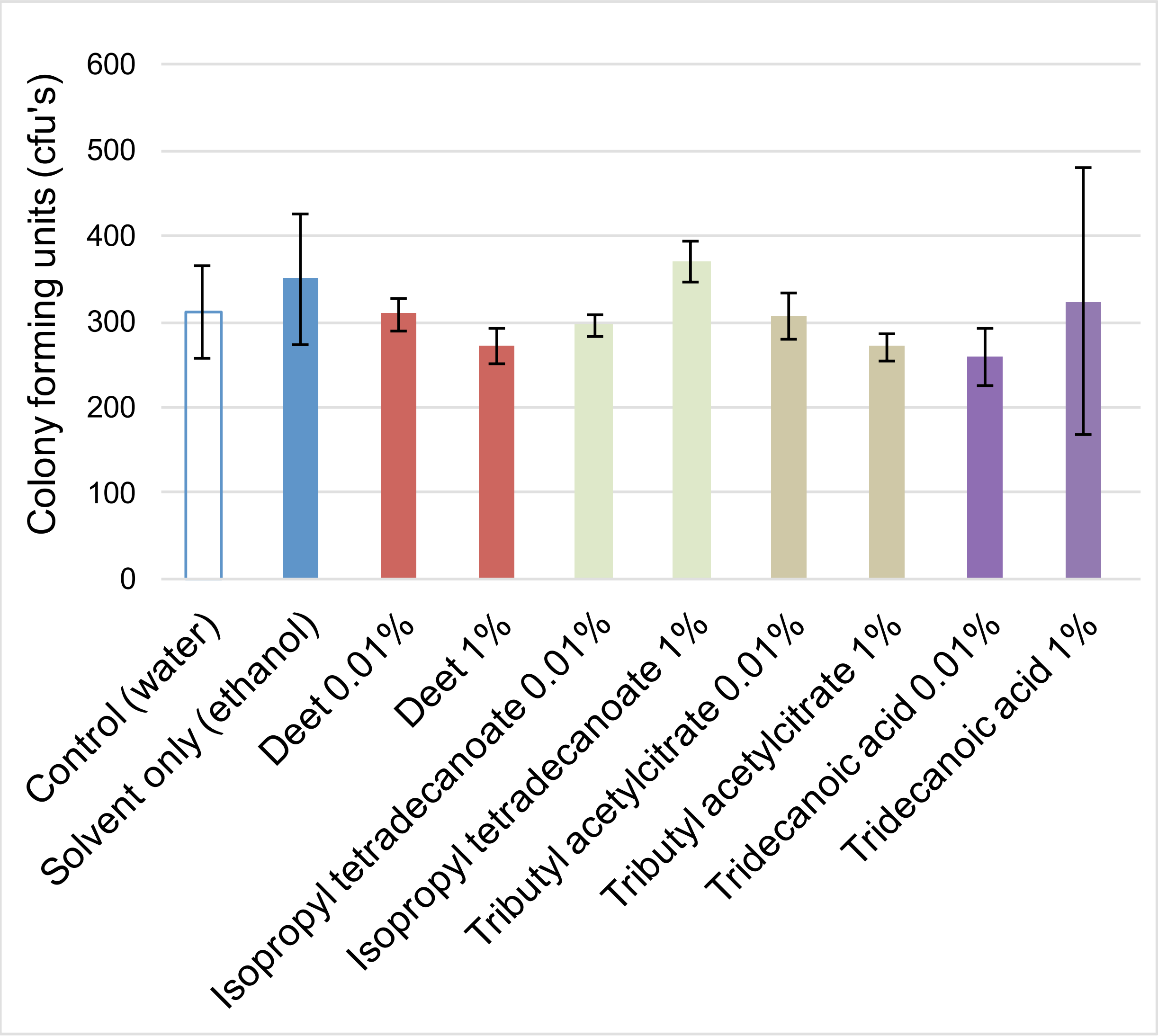

Supplementary Table S1. Mosquito response to the volatiles from different body parts. Individuals were not allowed to shower and use fragranced products for 24 hours before sampling. The volatiles of each body part were tested against a control of Ammonia in a dual-choice olfactometer.

		Released	Relative attractiveness % (RA)		Response %	
			Mean	Standard Error	Mean	Standard Error
Bodypart	Armpit	1310	53.02	4.28	30.04	2.38
	Foot	1307	66.24	3.69	34.66	2.99
	Hand	1331	59.88	4.38	37.90	3.07
Volunteer	1	426	58.11	8.28	36.21	6.35
	2	517	57.74	7.30	34.52	4.68
	3	486	78.16	4.57	35.51	5.24
	4	513	51.32	6.15	35.90	4.52
	5	494	37.88	6.19	26.67	3.69
	6	511	50.26	6.99	30.07	3.40
	7	495	72.73	5.02	40.01	5.18
	8	506	71.82	5.17	35.05	4.30

Supplementary Table S2. Mosquito response to volatiles from different body parts tested by direct comparison. Individuals were not allowed to use fragranced products for five days before sampling. The volatiles of each body part were tested directly against each other for each volunteer in a dual-choice olfactometer.

	Released	Proportion caught %			Response %	
	Sum	Mean	Mean	Standard Error	Mean	Standard Error
Foot *vs* Armpit	1384	Foot 50.3	Armpit 49.7	4.2	52.0	4.2
Hand *vs* Armpit	1383	Hand 57.8	Armpit 42.2	4.1	52.9	3.9
Hand *vs* Foot	1389	Hand 57.8	Foot 42.2	3.9	51.2	3.9
